# Active Usage of Mobile Health Applications: Cross-sectional Study

**DOI:** 10.2196/25330

**Published:** 2021-12-22

**Authors:** Yang Wang, Tailai Wu, Zhuo Chen

**Affiliations:** 1 College of Management Shenzhen University Shenzhen China; 2 School of Medicine and Health Management Huazhong University of Science and Technology Wuhan China; 3 Department of Health Policy and Management College of Public Health University of Georgia Athens, GA United States

**Keywords:** active usage, mobile health, 3-factor theory, consumer satisfaction, consumer dissatisfaction, medical informatics

## Abstract

**Background:**

Mobile health applications are being increasingly used for people’s health management. The different uses of mobile health applications lead to different health outcomes. Although active usage of mobile health applications is shown to be linked to the effectiveness of mobile health services, the factors that influence people’s active usage of mobile health applications are not well studied.

**Objective:**

This paper aims to examine the antecedents of active usage of mobile health applications.

**Methods:**

Grounded on the 3-factor theory, we proposed 10 attributes of mobile health applications that influence the active usage of mobile health applications through consumers’ satisfaction and dissatisfaction. We classified these 10 attributes into 3 categories (ie, excitement attributes, performance attributes, and basic attributes). Using the survey method, 494 valid responses were collected and analyzed using structural equation modeling.

**Results:**

Our analysis results revealed that both consumer satisfaction (β=0.351, *t*=6.299, *P*<.001) and dissatisfaction (β=–0.251, *t*=5.119, *P*<.001) significantly influenced active usage. With regard to the effect of attributes, excitement attributes (β=0.525, *t*=12.861, *P*<.001) and performance attributes (β=0.297, *t*=6.508, *P*<.001) positively influenced consumer satisfaction, while performance attributes (β=–0.231, *t*=3.729, *P*<.001) and basic attributes (β=–0.412, *t*=7.132, *P*<.001) negatively influenced consumer dissatisfaction. The results of the analysis confirmed our proposed hypotheses.

**Conclusions:**

Our study provides a novel perspective to study the active usage of mobile health applications. By categorizing the attributes of mobile health applications into 3 categories, the differential effects of different attributes can be tested. Meanwhile, consumer satisfaction and dissatisfaction are confirmed to be independent from each other.

## Introduction

### Background

With the introduction of smartphone devices in the world, mobile health applications are being increasingly used by consumers. By 2019, more than 2.5 billion people owned smartphones worldwide [1], while more than 50% of them had installed mobile health applications in their smartphones by 2017 [2]. The total market size of mobile health applications worldwide was forecasted to reach nearly US $100 billion in 2021 [3]. In the United States, more than 60% of patients use mobile health applications and other digital devices to manage their health [4], while in China, the number of active users of the most popular mobile health applications was over 10.5 million in January 2020 [5]. The most common mobile health applications were related to the weather, fitness, and nutrition in the United States [6] and healthy living information, measuring/recording of vital signs, and health and medical reminders in China by 2017 [7]. Therefore, mobile health applications have become an important component of individual health management.

Mobile health applications are internet-based applications in mobile devices to support medical and health activities [8]. Generally, mobile health applications provide functions to monitor consumers’ health status, record consumers’ health-related data, provide medical references, and assist in medical decision making [9]. Mobile health has been shown to affect consumers’ health behaviors, including physical activity, diet, alcohol, sexual behavior, and medication adherence [10]. Meanwhile, mobile health has been used to help manage health conditions, including diabetes, asthma, depression, hearing loss, anemia, and migraine [11]. However, the market of mobile health applications is still developing, even with their recognized benefits [12]. In addition to the effect of mobile health applications on people’s health status, the usage of mobile health applications affects the success of mobile health services [13].

According to usage status, mobile health application usage could be classified into active and passive. Active usage refers to consumers’ high engagement and complete usage of mobile health applications, while passive usage is the occasional and limited use of mobile health applications [14]. Compared with passive usage, active usage of mobile health applications not only uses different functions, such as posting and updating personal information or using nontraditional functions, but also has different antecedents. Since active usage of mobile health applications is linked to the effectiveness of mobile health services, including improving psychological flexibility [15], promoting the maintenance of health-related changes [16], and improving wellness [17], it is meaningful to study the active usage of mobile health applications.

The previous literature has studied the adoption or use of mobile health applications or services based on information system acceptance theories, such as the technology acceptance model (TAM), the theory of reasoned action (TRA), the theory of planned behavior (TPB), and the unified theory of acceptance and use of technology (UTAUT). For example, Hoque and Sorwar [18] extended UTAUT by incorporating technology anxiety and resistance to change in the study of mobile health adoption among older adults and found that only facilitating conditions do not have a significant effect. Alam et al [19] extended UTAUT with perceived reliability and price value and considered gender’s moderating role to study mobile health adoption and found that effort expectancy and price value do not influence adoption intention. A comprehensive literature review of mobile health adoption and usage is presented in Multimedia Appendix 1. However, few previous studies have considered the impact of mobile health application attributes and examined the factors that influence the active usage of mobile health applications. Since few prior studies on mobile health applications have examined active usage, we also consider the literature on active use or usage in other contexts. For example, Wu et al [20] studied the active usage of mobile instant messaging applications from an attachment perspective and categorized the predictors into 3 aspects: symbolism, aesthetics, and necessity. Davenport et al [21] found that narcissism is significantly related to active usage in college students but not in adults. A comprehensive literature review of active usage of information systems is presented in Multimedia Appendix 2. Although the previous literature has found several predictors of active usage, none of the predictors reflect the context of mobile health. Considering the importance of active usage of mobile health applications, this study seeks to understand and address the specific research question *What factors influence the active usage of mobile health applications?* Accordingly, we ground our research on the 3-factor theory and propose that consumer satisfaction and dissatisfaction could serve as the mechanisms of the effect of the factors on the active usage of mobile health applications. With regard to the factors, we mainly focus on the attributes of mobile health applications.

By addressing this research question, our study makes 3 important contributions: (1) We explore the factors of active usage of mobile health applications, which has largely been understudied; (2) we categorize and link the attributes to the active usage of mobile health applications based on the 3-factor theory; and (3) we extend and validate the 3-factor theory in the context of mobile health. The rest of the paper is organized as follows: In the next section, we review the literature on the 3-factor theory and consumer satisfaction/dissatisfaction, followed by our model and hypotheses. The subsequent section provides the research methodology and data analysis. Next, the results of the analysis and implications of our study for research and practice are discussed. We conclude with the limitations of this study and avenues for future research in the last section.

### Theoretical Background

In this study, the 3-factor theory was used as the overarching theory to construct the research model. Meanwhile, we discuss the theoretical background of satisfaction and dissatisfaction in this section.

#### 3-Factor Theory

The 3-factor theory is the extension of the 2-factor theory, originally proposed to explain job satisfaction in an organizational context [22]. However, the 2-factor theory was criticized for its oversimplification of influential factor categories and no context consideration [23]. Kano et al [24] refined the 2-factor theory and formulated the 3-factor theory to categorize product qualities into 3 factors that meet consumers’ needs. The 3 factors are termed as basic factors, excitement factors, and performance factors, aggregating 5 attributes of quality. In Kano’s model, basic factors correspond to hygiene factors in the 2-factor theory and represent consumers’ basic requirements, which do not affect satisfaction. Excitement factors correspond to motivation factors, which cause excitement but do not cause dissatisfaction when not present. The third category, termed “performance factors,” lies between basic and excitement factors, which cause satisfaction when present and dissatisfaction when not present.

The 3-factor theory has been widely applied in different contexts. In the previous literature, the 3-factor theory has been used to categorize online shopping website design attributes [25], test information systems qualities [26], and sort telecom service attributes [27]. Therefore, previous studies have demonstrated the validity and power of the 3-factor theory in examining the factors influencing usage and adoption and can provide a comprehensive understanding of the antecedents of mobile health application usage.

#### Consumer Satisfaction and Dissatisfaction

With regard to the relationship between consumer satisfaction and dissatisfaction, previous research has proposed 2 distinct views of the dimensionality of consumer satisfaction [21,28,29]. One view assumes that consumer satisfaction is unidimensional. Thus, consumer satisfaction and dissatisfaction are the 2 ends on a continuum. Another view postulates that consumer satisfaction is bidimensional. Therefore, consumer satisfaction and dissatisfaction are 2 independent constructs. In this study, we argue that consumer satisfaction and dissatisfaction are 2 separate constructs that follow a bidimensional view. The reason consumer satisfaction is not just opposite to consumer dissatisfaction in the current context is that consumer satisfaction and consumer dissatisfaction may also be caused by different factors, which are suggested by the 3-factor theory [23]. Some of the antecedent factors have a significant effect on consumer satisfaction but do not affect consumer dissatisfaction. Meanwhile, consumers’ satisfaction levels may not correspond to their dissatisfaction levels. Therefore, consumer satisfaction and dissatisfaction could coexist at the same time [27].

The previous literature also provides evidence that consumer satisfaction is bidimensional. Babin and Griffin [30] demonstrated that the 2-factor model of satisfaction and dissatisfaction could have an acceptable goodness of fit, which indicates that satisfaction and dissatisfaction are distinct. Chen et al [27] found that consumer satisfaction and consumer dissatisfaction have within-construct convergence and between-construct discriminant validities. Kim et al [31] found that factors linked to satisfaction and dissatisfaction of full-service hotels are distinct. Therefore, it is feasible to apply a bidimensional view of consumer satisfaction in our study.

#### Research Model and Hypothesis Development

**Figure 1:**
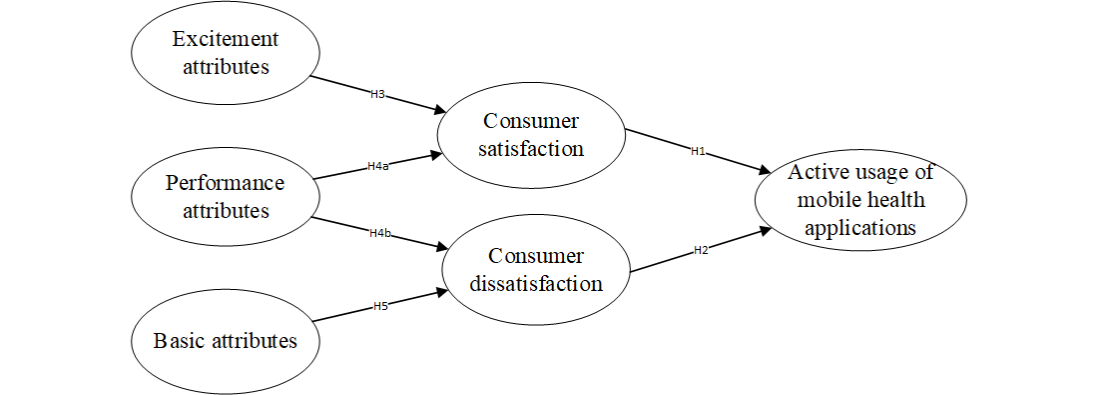
Research model.

According to the 3-factor theory, we proposed that the attributes of mobile health applications will result in satisfaction or dissatisfaction with the applications. Such satisfaction and dissatisfaction may lead to active usage of mobile health applications. Meanwhile, we classified the attributes of mobile health applications into basic attributes, excitement attributes, and performance attributes. The research model is presented in Figure 1.

#### Effect of Consumer Satisfaction and Dissatisfaction

According to the process view, consumer satisfaction refers to the assessment of whether the actual performance of a product or service conforms to one’s expectations [32,33]. When the actual performance of a product or service matches or goes beyond consumers’ expectations, they feel satisfied. Once they feel satisfied, they improve their attitudes toward the product or service [34,35], engage in positive word-of-mouth activities [36], and increase commitment [37].

Since active usage of mobile health applications can be a reflection of a strong commitment to the relationship between consumers and mobile health applications [38], it could be considered the behavioral representation of consumer engagement in using mobile health applications. Therefore, when consumers feel satisfied using a mobile health application, they will commit and engage in using that mobile health application. Hence, we hypothesize:

Hypothesis 1 (H1): Consumer satisfaction positively affects consumers’ active usage of mobile health applications.

Consumer dissatisfaction reflects a consumer’s evaluation process during nonconformation of the consumer’s expectations and the actual performance of products or services [39]. The previous literature reveals that consumer dissatisfaction might lead to negative behaviors, such as switching [40], complaining [41], or participating in negative word-of-mouth communication [36]. Nonconformity between consumers’ expectations and the actual performance of mobile health applications result in negative emotions, such as regret and disappointment [42]. Such negative emotions may only lead to poor commitment of the consumer at best toward using mobile health applications. Hence, we hypothesize:

H2: Consumer dissatisfaction negatively affects consumers’ active usage of mobile health applications.

#### Attributes of Mobile Health Applications

To identify the attributes that influence consumers’ active usage of mobile health applications, we conducted interviews as well as a thorough literature review. First, we conducted open-ended interviews with 50 mobile health application users in China by asking them questions such as *Which attributes of mobile health applications influence you to use them actively?* We recorded the responses regarding satisfaction/dissatisfaction in the form of keywords and short sentences. Two PhD students then conducted the content analysis of the recorded responses to identify the items that may reflect the attributes of mobile health applications. Disagreements between them were resolved through discussion. Second, we screened the literature on mobile health adoption to identify possible attributes and established an attribute pool by combining the attributes from the interviews and our thorough literature review. We then selected attributes based on whether they were applicable in mobile health applications. The definitions of the attributes and their sources are summarized in Table 1. Finally, we divided the attributes into 3 categories based on the 3-factor theory.

**Table 1:** Summary of mobile health applications’ attributes.

Number	Attribute	Definition	Source
1	Design aesthetics	The degree of attractiveness or beauty of a mobile health application’s interface	Lavie and Tractinsky [43]
2	Customization	The degree to which mobile health application providers tailor their products for different consumers	Srinivasan et al [44]
3	Enjoyment	The degree of fun or pleasure consumers get from using mobile health applications	Van der Heijden [45]
4	Mobility	The degree to which consumers can use mobile health applications regardless of location and time	Hong et al [46]
5	Sociability	The extent to which mobile health applications support social interaction between consumers	Preece [47]
6	Informational support	The degree to which mobile health applications provide information for solving consumers’ health concerns	Liang et al [48]
7	Emotional support	The degree to which doctors and other consumers convey their care and understanding to consumers through mobile health applications	Liang et al [48]
8	Perceived security	The extent of security of consumers’ private and sensitive information in using mobile health applications	Chang and Chen [49]
9	Technical functionality	Consumers’ assessment of mobile health application accessibility, stability, response time, and operation	Ou and Sia [25]
10	Information quality	Consumers’ assessment of the accuracy, relevance, and timeliness of information generated by mobile health applications	Delone and Mclean [50]

Combining the definitions of specific attributes with the definitions of the 3 categories, we could categorize the identified attributes into those 3 categories. According to the 3-factor theory, excitement and performance attributes would cause consumer satisfaction, while performance and basic attributes would drive consumer dissatisfaction. With the presence of different attributes, consumer satisfaction and dissatisfaction may coexist. To be specific, we categorized design aesthetics, customization, and enjoyment as excitement attributes because the 3 specific attributes are not the primary concern of consumers but bring additional value to them. Next, we categorized sociability, informational support, and emotional support as performance attributes because these specific attributes can facilitate consumer interaction and communication with health professionals, which are consumers’ main purposes of using mobile health applications. Finally, we categorized mobility, perceived security, technical functionality, and information quality as basic attributes because these specific attributes are the basic requirements of any mobile application [51]. This categorization was also preliminarily confirmed by using a card-sorting method [52]. In this study, we treated excitement, performance, and basic attributes as second-order formative constructs that contain the attributes proposed by us and confirmed by the card-sorting method. The relationship between the attributes and consumer satisfaction/dissatisfaction is specified next.

With regard to excitement attributes, design aesthetics, such as the choice of color, shape, or layout of mobile health applications, decide consumers’ aesthetics [43]. Meanwhile, design aesthetics can fulfill people’s needs for aesthetics in using mobile health applications [53]. Therefore, design aesthetics may influence consumers’ satisfaction from using mobile health applications. Customization increases consumers’ perceived control toward a mobile health application and signals the quality of the mobile health application [54]. The perceived control and high quality of mobile health applications can influence consumers’ satisfaction from using the mobile health applications. With regard to enjoyment, providing consumers with an enjoyable experience is critical for influencing their perception of mobile health applications [45]. Enjoyment in using a mobile health application would be an intrinsic motivation for consumers in using the mobile health application [55]. Therefore, enjoyment reflects the satisfaction of consumers’ intrinsic motivation. Hence, we hypothesize:

H3: Excitement attributes, which are characterized by design aesthetics, customization, and enjoyment, positively affect consumers’ satisfaction.

With regard to performance attributes, mobile health applications integrate various forms and functions to support the communication and interaction among consumers [47]. Facilitating interaction between health professionals and consumers produces a flow experience in using mobile health applications and increases consumers’ satisfaction from using mobile health applications [56]. If mobile health applications cannot facilitate interaction, a consumer may be dissatisfied. Therefore, sociability may lead to satisfaction if mobile health applications have this attribute and dissatisfaction if mobile health applications do not have it. Meanwhile, informational support and emotional support are both dimensions of social support. Health information from mobile health applications could satisfy consumers’ needs to deal with their health problems. In contrast, emotional support from doctors and other consumers using mobile health applications might provide emotional support and meet consumers’ emotional needs [57]. If mobile health applications cannot afford informational support and emotional support, consumers’ health needs may not be satisfied and the evoked negative emotions may make them feel dissatisfied with using mobile health applications. Summarizing the reasonings above, we hypothesize:

H4a: Performance attributes, which are characterized by sociability, informational support, and emotional support, positively affect consumer satisfaction.

H4b: Performance attributes, which are characterized by sociability, informational support, and emotional support, negatively affect consumer dissatisfaction.

With regard to mobility, a basic attribute, which enables consumers to use mobile health applications anywhere and anytime, does not just belong to mobile health applications but also to mobile devices [58]. Given that mobility is shared by other applications on mobile devices [59], it could be considered the basic factor behind using mobile health applications. Therefore, mobility may lead to dissatisfaction if mobile health applications do not have this attribute. Perceived security is 1 of the key drivers for building trust to use any IT [49]. Although some mobile health applications do not integrate mobile shopping functions, and consumers do not suffer any monetary loss, consumers’ private information may still be subject to unauthorized access, use, storage, or transmission. Consumers may require application providers to ensure the security of their private information in using mobile health applications. Therefore, perceived security is the basic attribute that may only affect consumers’ dissatisfaction. With regard to technical functionality, the technical functions of mobile health applications, such as response speed, stability, and accessibility, may define consumers’ usage experience [60], but these functions are not only specific to mobile health applications but also crucial for other IT artifacts. Therefore, technical functionality is a basic attribute and may only influence consumer dissatisfaction if this attribute does not perform well. Regarding information quality, the 3 main aspects that define it are relevance, timeliness, and accuracy of the transmitted information [61]. The quality of communication from using a mobile health application depends upon the quality of information that is transmitted through the mobile health application [62]. Therefore, information quality also serves as the basic condition to complete the main purposes of using mobile health applications and leads to consumer dissatisfaction if it is absent. Hence, we hypothesize:

H5: Basic attributes, which are characterized by mobility, perceived security, technical functionality, and information quality, negatively affect consumer dissatisfaction.

## Methods

### Measurement Instrument

The survey method was used in this study to validate our proposed research model. The measurement instrument was developed by adapting previously validated scales. The constructs and items sources are presented in Table 2. All items were measured on a 7-point Likert scale with anchors ranging from 1=strongly disagree to 7=strongly agree. In addition, our study included several control variables that measured consumers’ characteristics, such as age, gender, education, length, and frequency of using mobile health applications. The length of using mobile health applications reflects how long users have been using mobile health applications, while the frequency of using mobile health applications indicates how many times users use mobile health applications per day.

**Table 2:** Instrument sources.

Construct	Source
Active usage	Pagani and Mirabello [63]
Satisfaction	Taylor and Baker [64]
Dissatisfaction	Babin and Griffin [30]
Design aesthetics	Lavie and Tractinsky [43]
Customization	Srinivasan et al [44]
Enjoyment	Sun and Zhang [65]
Sociability	Animesh et al [56]
Mobility	Hong et al [46]
Informational support	Liang et al [48]
Emotional support	Liang et al [48]
Information quality	Ou and Sia [25]
Technical functionality	Ou and Sia [25]
perceived security	Cheung and Lee [66]

Since this study was conducted in China and the respondents were primarily Chinese, we translated the survey into Chinese using the back-translation method. The English instrument was first translated into Chinese by 1 bilingual author. Next, another bilingual author back-translated the Chinese version into English. The 2 authors then compared the 2 English versions for inconsistencies. Next, 8 experts of health information systems and 16 users of mobile applications were interviewed to identify ambiguous or repetitive items and suggestions obtained to improve the quality of the survey instrument. Finally, we revised the questionnaire according to the comments and suggestions received. The details of the survey instrument are presented in Multimedia Appendix 3.

### Data Collection

The data were collected in China, which is 1 of the largest mobile markets in the world [67]. The online survey was conducted by using a paid service from a popular web survey company in China. The institutional review board of Tongji Medical College, Huazhong University of Science and Technology, China, approved our study procedures (no. 2017S319). Through a 3 weeks’ survey in May 2020, we sent the questionnaire to 782 users of Chinese mobile health application users through the online survey company randomly in their users’ pool and obtained a total of 674 responses. Therefore, the response rate of our survey was 86.2%.

Given we used an online survey, possible issues, including convenience sample, superrespondents, abnormal respondents, or common method bias, might have existed. By following the guidelines of online surveys, we took several actions that are recommended to ensure data quality [68]. First, our choice of a paid service of online survey company could deal with the issue of a convenience sample since the company has users with diverse backgrounds in different areas. Second, we set screening questions to ask whether the respondents were actual mobile health application users, eliminating respondents who may have produced irrelevant responses. The screening questions included *Do you have mobile health applications in your smartphone or tablet?* *How many mobile health applications have you installed on your smartphone or tablet?* Actual users could be identified by checking whether they were using mobile health applications according to their answers to these screening questions. Third, to address the issue of superrespondents who were good at filling online questionnaires, we eliminated responses with an unreasonably short time (less than 5 minutes) to finish the questionnaire. Fourth, we eliminated responses where respondents did not correctly answer reverse-coded and attention-trap questions and gave too many same answers for all questions (more than 90%) in order to eliminate haphazard responses. A reverse-coded question is negative worded, and its score needs to be reversed for further data analysis, while an attention-trap question is easy to be answered with only 1 correct option. Finally, we conducted procedural remedies to deal with the common method bias, including randomizing items and using different response formats [57].

After cleaning the collected data, we were left with 494 complete and valid responses. In this sample, most of the respondents were in the age group of 18-30 years, were female (280/494, 56.7%), possessed a college degree, and were familiar with mobile health applications. The demographic distribution of the sample in our study is consistent with the China Internet Network Information Center report of the national profile of mobile internet users [69]. The specific demographic information of our final sample is summarized in Table 3.

**Table 3:** Demographic information (N=494).

Characteristics		n (%)
**Age (years)**		
	18-25	151 (30.6)
	25-30	222 (44.9)
	>30	121 (24.5)
**Gender**		
	Male	214 (43.3)
	Female	280 (56.7)
**Education**		
	High school	14 (2.8)
	College	414 (83.8)
	Master degree and above	66 (13.4)
**Length of using mobile health applications (years)**		
	<1	11 (2.3)
	1-3	222 (44.9)
	>3	261 (52.8)
**Frequency of using mobile health applications**		
	Almost 1 time/day	22 (4.5)
	1-10 times/day	280 (56.7)
	>10 times/day	192 (38.8)

### Data Analysis

This study used partial least squares (PLS), a technique of structural equation modeling, to analyze the data. PLS is a second-generation multivariate causal analysis method and can analyze complex structural equation models [70]. PLS can also be applied in exploratory studies and aims at theory building rather than theory testing. In addition, PLS can model constructs with either formative or reflective indicators. The analysis was conducted using SmartPLS 2.0.3M (SmartPLS GmbH) [71]. Following the 2-stage approach suggested by Anderson and Gerbing [72], we analyzed the measurement model to test reliability and validity, followed by the analysis of a structural model to test our research model. To test reliability and validity, confirmatory factor analysis was conducted, while we implemented a bootstrapping procedure with PLS to analyze the structural model. The results of reliability and validity of our developed measurement instrument and structural model are presented in the Results section.

## Results

### Reliability and Validity

The indicators of reliability and validity are summarized in Tables 4-6. In Table 4, all Cronbach α and composite reliabilities are >.7, thus demonstrating reliability for all constructs. The value of the average variance extracted (AVE) of each construct was >0.5, thus demonstrating good convergent validity [73]. Based on the results in Tables 5 and 6, each item loading for its assigned construct was >0.7 and even higher for other constructs, while the square roots of the AVEs were all greater than the interconstruct correlations, thus demonstrating discriminant validity [74]. Hence, we concluded that the quality of the measurement model is adequate for testing hypothesized relationships.

**Table 4:** Construct reliability and convergent validity.

Construct	Composite reliability	AVE^a^	Cronbach α
Active usage	0.84	0.64	.71
Satisfaction	0.92	0.80	.88
Dissatisfaction	0.94	0.84	.91
Design aesthetics	0.93	0.72	.90
Customization	0.95	0.90	.89
Enjoyment	0.92	0.79	.87
Mobility	0.91	0.71	.86
Sociability	0.89	0.74	.82
Information quality	0.89	0.80	.75
Informational support	0.91	0.84	.80
Emotional support	0.91	0.83	.79
Perceived security	0.96	0.92	.91
Technical functionality	0.88	0.72	.80

^a^AVE: average variance extracted.

**Table 5:** Loading and cross-loading.

Construct	Active usage	Consumer satisfaction	Consumer dissatisfaction	Design aesthetics	Customization	Enjoyment	Mobility	Sociability	Information quality	Informational support	Emotional support	Perceived security	Technical functionality
Active usage 1	*0.82* ^a^	0.40	–0.37	0.32	0.13	0.39	0.43	0.41	0.36	0.31	0.30	0.14	0.30
Active usage 2	*0.84* ^a^	0.44	–0.39	0.35	0.18	0.43	0.43	0.42	0.37	0.35	0.37	0.25	0.35
Active usage 3	*0.73* ^a^	0.38	–0.36	0.43	0.25	0.40	0.35	0.38	0.35	0.30	0.25	0.19	0.31
Consumer satisfaction 1	0.45	*0.89* ^a^	–0.55	0.54	0.30	0.59	0.47	0.52	0.49	0.42	0.37	0.36	0.44
Consumer satisfaction 2	0.46	*0.90* ^a^	–0.57	0.58	0.35	0.67	0.45	0.55	0.53	0.41	0.33	0.39	0.43
Consumer satisfaction 3	0.45	*0.89* ^a^	–0.56	0.57	0.31	0.60	0.39	0.50	0.45	0.38	0.34	0.36	0.42
Consumer dissatisfaction 1	–0.44	–0.56	*0.92* ^a^	–0.47	–0.30	–0.48	–0.43	–0.42	–0.44	–0.32	–0.25	–0.35	–0.41
Consumer dissatisfaction 2	–0.44	–0.58	*0.91* ^a^	–0.47	–0.31	–0.48	–0.43	–0.47	–0.44	–0.35	–0.34	–0.36	–0.41
Consumer dissatisfaction 3	–0.41	–0.58	*0.92* ^a^	–0.50	–0.27	–0.50	–0.42	–0.44	–0.42	–0.37	–0.34	–0.39	–0.42
Design aesthetics 1	0.41	0.52	–0.42	*0.85* ^a^	0.43	0.53	0.44	0.46	0.54	0.41	0.31	0.39	0.45
Design aesthetics 2	0.42	0.53	–0.48	*0.85* ^a^	0.40	0.57	0.42	0.46	0.52	0.39	0.37	0.42	0.46
Design aesthetics 3	0.33	0.50	–0.37	*0.82* ^a^	0.45	0.50	0.30	0.43	0.50	0.33	0.18	0.46	0.36
Design aesthetics 4	0.39	0.53	–0.38	*0.85*	0.46	0.52	0.33	0.45	0.52	0.29	0.20	0.44	0.40
Design aesthetics 5	0.38	0.57	–0.52	*0.86* ^a^	0.41	0.62	0.43	0.48	0.54	0.37	0.29	0.46	0.50
Customization 1	0.21	0.31	–0.29	0.47	*0.94* ^a^	0.33	0.21	0.32	0.41	0.11	0.03	0.46	0.26
Customization 2	0.23	0.36	–0.31	0.49	*0.96* ^a^	0.38	0.25	0.36	0.44	0.16	0.09	0.52	0.30
Enjoyment 1	0.47	0.64	–0.51	0.60	0.34	*0.89* ^a^	0.48	0.53	0.53	0.41	0.28	0.45	0.46
Enjoyment 2	0.44	0.61	–0.46	0.58	0.32	*0.90* ^a^	0.44	0.51	0.48	0.42	0.34	0.39	0.42
Enjoyment 3	0.45	0.60	–0.44	0.55	0.32	*0.88* ^a^	0.40	0.46	0.45	0.36	0.29	0.34	0.40
Mobility 1	0.42	0.40	–0.39	0.38	0.20	0.43	*0.85* ^a^	0.45	0.43	0.34	0.32	0.20	0.49
Mobility 2	0.41	0.40	–0.37	0.35	0.13	0.41	*0.81* ^a^	0.44	0.37	0.44	0.39	0.19	0.41
Mobility 3	0.44	0.39	–0.39	0.43	0.26	0.38	*0.83* ^a^	0.48	0.44	0.34	0.29	0.21	0.48
Mobility 4	0.43	0.45	–0.40	0.39	0.22	0.44	*0.87* ^a^	0.47	0.47	0.37	0.39	0.19	0.53
Sociability 1	0.46	0.52	–0.44	0.50	0.35	0.52	0.45	*0.86* ^a^	0.50	0.36	0.30	0.33	0.44
Sociability 2	0.43	0.51	–0.43	0.48	0.32	0.47	0.48	*0.88* ^a^	0.51	0.40	0.33	0.36	0.43
Sociability 3	0.42	0.47	–0.38	0.40	0.26	0.46	0.47	*0.84* ^a^	0.45	0.32	0.35	0.30	0.44
Information quality 1	0.40	0.49	–0.43	0.53	0.42	0.48	0.46	0.52	*0.89* ^a^	0.36	0.23	0.45	0.50
Information quality 2	0.40	0.50	–0.41	0.58	0.38	0.50	0.45	0.49	*0.89* ^a^	0.43	0.31	0.40	0.55
Informational support 1	0.39	0.42	–0.34	0.38	0.11	0.43	0.41	0.40	0.39	*0.91* ^a^	0.52	0.24	0.39
Informational support 2	0.35	0.40	–0.36	0.40	0.15	0.38	0.40	0.37	0.41	*0.91* ^a^	0.48	0.22	0.41
Emotional support 1	0.34	0.37	–0.31	0.29	0.05	0.33	0.36	0.35	0.27	0.51	*0.92* ^a^	0.11	0.40
Emotional support 2	0.36	0.33	–0.30	0.30	0.07	0.30	0.39	0.34	0.28	0.48	*0.90* ^a^	0.12	0.36
Perceived security 1	0.21	0.40	–0.37	0.48	0.50	0.43	0.20	0.37	0.45	0.23	0.12	*0.96* ^a^	0.33
Perceived security 2	0.26	0.39	–0.39	0.50	0.49	0.42	0.25	0.37	0.47	0.26	0.12	*0.96* ^a^	0.33
Technical functionality 1	0.34	0.43	–0.36	0.43	0.27	0.40	0.51	0.45	0.47	0.37	0.35	0.30	*0.82* ^a^
Technical functionality 2	0.32	0.36	–0.36	0.42	0.22	0.39	0.48	0.41	0.52	0.38	0.33	0.29	*0.85* ^a^
Technical functionality 3	0.36	0.42	–0.43	0.46	0.27	0.43	0.47	0.42	0.51	0.37	0.39	0.29	*0.87* ^a^

^a^The square roots of the average variances extracted (AVEs) are in italic.

**Table 6:** Discriminant validity of constructs.

Construct	Active usage	Consumer satisfaction	Consumer dissatisfaction	Design aesthetics	Customization	Enjoyment	Mobility	Sociability	Information quality	Informational support	Emotional support	Perceived security	Technical functionality
Active usage	*0.* *80* ^a^	—^b^	—	—	—	—	—	—	—	—	—	—	—
Consumer satisfaction	0.51	*0.90* ^a^	—	—	—	—	—	—	—	—	—	—	—
Consumer dissatisfaction	–0.47	–0.62	*0.92* ^a^	—	—	—	—	—	—	—	—	—	—
Design aesthetics	0.46	0.63	–0.52	*0.85* ^a^	—	—	—	—	—	—	—	—	—
Customization	0.23	0.35	–0.32	0.51	*0.95* ^a^	—	—	—	—	—	—	—	—
Enjoyment	0.51	0.70	–0.53	0.65	0.37	*0.89* ^a^	—	—	—	—	—	—	—
Mobility	0.51	0.49	–0.46	0.46	0.25	0.50	*0.84* ^a^	—	—	—	—	—	—
Sociability	0.51	0.58	–0.48	0.54	0.36	0.56	0.55	*0.86* ^a^	—	—	—	—	—
Information quality	0.45	0.55	–0.47	0.62	0.45	0.55	0.51	0.57	*0.* *89* ^a^	—	—	—	—
Informational support	0.40	0.45	–0.38	0.42	0.14	0.45	0.44	0.42	0.44	*0.91* ^a^	—	—	—
Emotional support	0.39	0.39	–0.34	0.32	0.06	0.34	0.41	0.38	0.30	0.55	*0.91* ^a^	—	—
Perceived security	0.25	0.41	–0.40	0.52	0.52	0.44	0.24	0.38	0.48	0.25	0.13	*0.96* ^a^	—
Technical functionality	0.40	0.48	–0.45	0.51	0.30	0.48	0.57	0.51	0.59	0.44	0.42	0.34	*0.8* *5* ^a^

^a^The square roots of average variances extracted (AVEs) are in italic.

^b^Not applicable.

We also examined the possibility of common method bias in our study. First, we investigated the correlation coefficients between variables in Table 4 and found that none of the pairs had a high correlation (*r*>.90) [75]. Second, we conducted the Harman single-factor test using principal component analysis in SPSS Statistics 18.0. In total, 10 factors were extracted, and the first factor in the unrotated solution explained 39.61% of the variation, which is <50% [76]. Third, we employed the marker variable technique to test common method bias [77]. Organizational commitment was chosen as the marker variable since it was theoretically unrelated to our research model. We found organizational commitment had no significant effect on the active usage of mobile health applications (β=.014, *P*>.05).

### Hypotheses Testing

**Figure 2:**
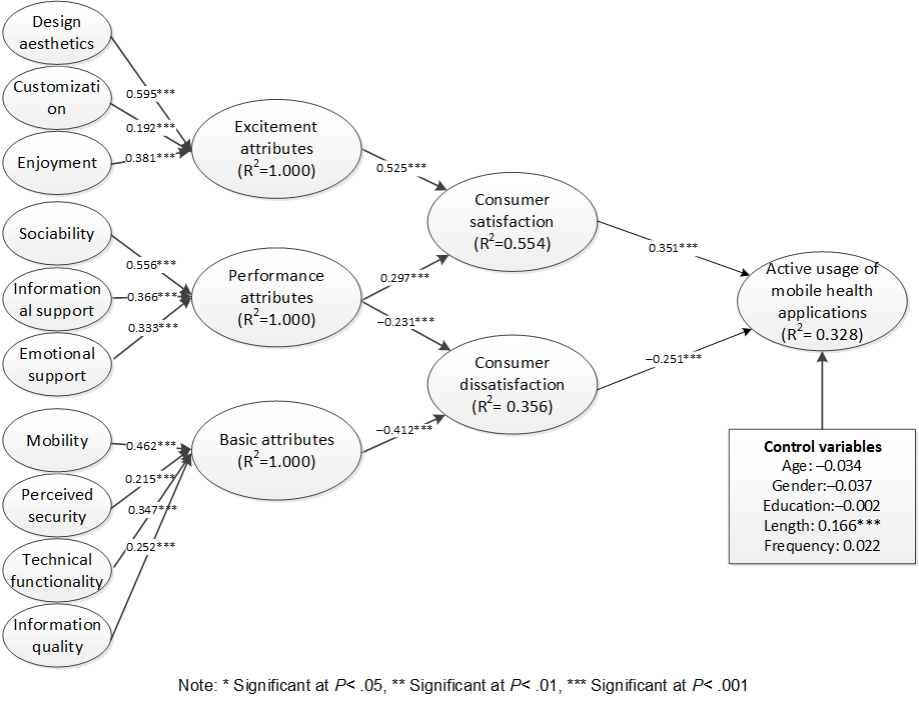
Structural model analysis results.

The descriptive statistics of variables in our research model are presented in Table 7. The results of structural model analysis are summarized in Figure 2. The results reveal that both consumer satisfaction (β=.351, *t*=6.299, *P*<.001) and dissatisfaction (β=–.251, *t*=5.119, *P*<.001) significantly influence consumers’ active usage of mobile health applications, thus supporting H1 and H2. This implies that the 3-factor theory is a useful theoretical perspective for predicting consumers’ active usage of mobile health applications.

**Table 7:** Descriptive statistics of variables.

Construct	Mean (SD)
Active usage	6.00 (0.83)
Satisfaction	5.90 (0.78)
Dissatisfaction	2.07 (0.80)
Design aesthetics	5.70 (0.86)
Customization	4.93 (1.20)
Enjoyment	5.91 (0.77)
Mobility	6.04 (0.84)
Sociability	5.95 (0.82)
Information quality	5.46 (1.06)
Informational support	6.12 (0.72)
Emotional support	6.39 (0.67)
Perceived security	5.21 (1.27)
Technical functionality	5.91 (0.81)

As a second-order formative construct, excitement attributes (β=.525, *t*=12.861, *P*<.001) were revealed to significantly influence consumer satisfaction. Therefore, H3 is supported. Moreover, the corresponding first-order constructs, including design aesthetics, customization, and enjoyment, together comprised excitement factors. With regard to performance attributes, the results also show that they influence both consumer satisfaction (β=.297, *t*=6.508, *P*<.001) and dissatisfaction (β=–.231, *t*=3.729, *P*<.001) significantly. Therefore, H4a and H4b are supported. Meanwhile, the corresponding first-order constructs, including sociability, informational support, and emotional support, are necessary components of performance factors. With regard to basic attributes (β=–.412, *t*=7.132, *P*<.001), which include mobility, perceived security, technical functionality, and information quality, they were found to have a significant influence over consumer dissatisfaction. Therefore, H5 is supported. These results showed that the categorization of basic factors is valid. Finally, the effect of control variables was considered. Only the length of using mobile health applications was significantly associated with active usage of mobile health applications.

## Discussion

### Principle Findings and Implications

In this study, we identified 10 attributes of mobile health applications that would influence people’s active usage. Based on the 3-factor theory, we divided the attributes into 3 categories to correspond to 3 factors, namely excitement attributes, performance attributes, and basic attributes. Excitement and performance attributes are assumed to significantly affect consumer satisfaction, while performance and basic attributes are supposed to significantly impact consumer dissatisfaction. Both consumer satisfaction and dissatisfaction would influence active usage of mobile health applications. Excitement attributes include design aesthetics, customization, and enjoyment; performance attributes include sociability, informational support, and emotional support; and basic attributes include mobility, perceived security, technical functionality, and information quality. To validate the categorization and the effect of attributes on active usage of mobile health applications, we used the survey method and analyzed the data using the PLS technique. The empirical results confirmed our hypothesized differential effects of the 3 attributes on consumer satisfaction and dissatisfaction and then on the active usage of mobile health applications. Meanwhile, all the first-order constructs were also found to be significantly linked to their corresponding second-order constructs. Therefore, the feasibility and validity of the 3-factor theory were manifested in our study. The interestingness and uniqueness of our findings in terms of theoretical and practical implications are further discussed.

From a theoretical perspective, we made several contributions. First, we contributed to health information system usage and adoption literature by studying the active usage of mobile health applications, thus uncovering insights into the use or adoption of mobile health applications. Since most studies do not consider active usage of mobile health applications, as was done in this paper [18,19], considering the importance of active usage toward the effectiveness of mobile health applications, the results of this paper provide a better and deeper understanding of mobile health application adoption and usage.

Second, we also contributed to health information system usage and adoption literature by identifying several influential attributes of active usage of mobile health and categorizing them based on the framework of the 3-factor theory. Previous literature focuses little on the effects of attributes of mobile health applications. This study not only identifies the underlying attributes but also distinguishes the different roles of various attributes of active usage of mobile health applications based on the 3-factor theory. For example, excitement and performance attributes can drive the active usage of mobile health applications, whereas performance and basic attributes could impede the active usage of mobile health applications. Moreover, the study results also reveal a valid categorization of identified attributes.

Third, we contributed to health information system usage and adoption literature by studying the active usage of mobile health applications. Previous studies of active usage of information systems have focused on mobile instant messaging [20], social media [21], or online communities [78-80], a few of them revealing factors driving active usage of mobile health applications. Therefore, our study enriches the literature of active usage of health information systems. At the same time, our study reflects the characteristics of the active usage of mobile health applications by exploring the unique attributes that lead to active usage, such as informational support and emotional support.

Finally, we contributed to the 3-factor theory literature by extending it to the context of mobile health applications. The previous literature mainly applies the 3-factor theory in contexts such as website design or telecom service [25,27], while our study validates the feasibility of the 3-factor theory in the mobile health context. Meanwhile, based on the 3-factor theory, we propose both satisfaction and dissatisfaction as mediators between the antecedents of the usage of mobile health applications and active usage of mobile health applications. Our result provides further support for the distinctiveness between satisfaction and dissatisfaction.

From the practical perspective, this study will help policymakers and medical providers identify active users by using our measurement items of active usage of mobile health applications. For example, the time spent in using mobile health applications, the usage frequency, and some traces of consumers’ usage behavior, such as the volume of consumer-generated content in mobile health applications, could be used to compose some indices to locate active users. The thresholds for spending time, usage frequency, generated content, or other tracks could be set to decide who are active users and who are passive users. After identifying the different usage status of different users, policymakers and medical providers could use corresponding strategies to promote the engagement of different users in using mobile health applications.

This study empirically identifies attributes that could be useful for predicting consumers’ active usage of mobile health applications and help policymakers and application designers promote proper mobile health applications for consumers. For example, to improve consumers’ usage, mobile health applications should focus on excitement attributes and performance attributes, including design aesthetics, customization, enjoyment, sociability, informational support, and emotional support on the premise that mobility, information quality, perceived security, and technical functionality of the mobile health applications are acceptable.

Finally, because consumer satisfaction and dissatisfaction both influence active usage, practitioners should not only emphasize consumer satisfaction in using mobile health applications but also reduce consumer dissatisfaction from using mobile health applications. Once a consumer feels dissatisfied with using any aspect of a mobile health application, they may become a passive user from being an active user. If they continue to remain dissatisfied with using the mobile health application, and the barriers to switching are low, then they may switch to other mobile health applications.

### Limitations and Future Research

The results of this study could be interpreted in light of its limitations. First, although we identified several antecedents of mobile health application usage, the explained variances of active usage of mobile health applications still have the potential to be improved. Our literature review of mobile health application usage and adoption revealed that social, personal, and motivational factors could influence mobile health application usage and adoption, whose effects on active usage of mobile health applications are worth to be examined. Future studies may examine other factors to better understand consumers’ active usage.

Second, the generalizability may be restricted because our sample was restricted to Chinese consumers. In China, the most popular mobile health application is Ping An Good Doctor [5], but in other countries, other mobile health applications, such as Pedometer in Sweden [78] or Samsung Health in the United States [79], are leading. There are differences between mobile health applications in different countries. Future studies may conduct cross-country comparisons to better generalize the results of this study.

Finally, our study was mainly a cross-sectional one, where constructs are measured at the same point of time. However, since consumer behavior and mobile health applications are both dynamic, the results may change over time. Therefore, the cross-sectional method may not reflect the dynamics of mobile health application usage. A longitudinal study with a multimethod approach may help address this issue.

### Conclusion

In this study, we explored the factors influencing consumers’ active usage of mobile health applications based on the 3-factor theory. According to the 3-factor theory, we focused on the attributes of mobile health applications and divided them into 3 categories: excitement, performance, and basic. The 3 categories of attributes are assumed to influence the active usage of mobile health applications through consumer satisfaction and dissatisfaction. The proposed relationships were validated by using a survey method. The analysis results not only imply that consumer satisfaction and dissatisfaction are independent from each other, but also confirm the categorization of attributes for active usage of mobile health applications. Meanwhile, our study could inspire designers and policymakers to make patients actively use mobile health applications.

## Appendix

Multimedia Appendix 1 Literature review of mobile health adoption and usage.

Multimedia Appendix 2 Literature review of active usage.

Multimedia Appendix 3 Survey instruments.

## Abbreviations

AVE: average variance extracted
PLS: partial least squares
TAM: technology acceptance model
TPB: theory of planned behavior
TRA: theory of reasoned action
UTAUT: unified theory of acceptance and use of technology
