# Supplementary material for: Active Usage of Mobile Health Applications: Cross-sectional Study
Source: J Med Internet Res. 2021 Dec 22;23(12):e25330. doi: 10.2196/25330 (PMC8734924; doi:10.2196/25330)
Supplement: Multimedia Appendix 1 [file jmir_v23i12e25330_app1.docx]

# Appendix 1. Literature review of mobile health adoption and usage

To understand the literature of mobile health adoption and usage systematically and comprehensively, we survey previous literature and present the result in table 1. We summarize previous literature from four aspects: authors, factors, theories and adoption/usage. According to table 1, previous literature explored the factors based on information systems acceptance theories like the Technology Acceptance Model (TAM), Theory of Reasoned Action (TRA), Theory of Planned Behavior (TPB), and Unified Theory of Acceptance and Use of Technology (UTAUT). Therefore, previous literature about mobile health adoption and usage pay less attention to the attributes of mobile health applications.

| **Table 1. Literature review of mobile health adoption and usage** | | | | |
| --- | --- | --- | --- | --- |
| No. | Authors | Factors | Theories | Adoption/usage |
| 1 | Guo et al. [1] | Technology anxiety, perceived ease of use, perceived usefulness, resistance to change | Dual factor model of technology acceptance | Preventive mobile health services adoption |
| 2 | Rai et al. [2] | Personal innovativeness, distance to health facilities, recent health check-up, perceived healthiness, perceived vulnerability. | N/A | Mobile health usage |
| 3 | Cocosila [3] | Perceived risk, extrinsic motivation, intrinsic motivation, attitude towards activity | Motivational model | Mobile health application adoption |
| 4 | Sun et al. [4] | Response efficacy, perceived ease of use, subjective norm, response cost, self-efficacy, perceived vulnerability, perceived severity | TAM, TRA, TPB, UTAUT, protection motivation theory. | Acceptance of mobile health services |
| 5 | Zhang et al. [5] | Facilitating conditions, attitude, subjective norms | TRA | m-Health adoption |
| 6 | Shareef et al. [6] | Perceived usefulness, perceived ease of use, perceived compatibility, perceived reliability, perceived privacy and security | TAM | m-Health adoption |
| 7 | Okazaki et al. [7] | Subjective norm, ubiquitous control, job relevance, information quality, health improvement, perceived value, ease of use | TAM | m-health usage |
| 8 | Dwivedi et al. [8] | Performance expectancy, effort expectancy, social influence, facilitating conditions, hedonic motivation, price value, habit, waiting time, self-concept | UTAUT | m-Health adoption |
| 9 | Guo et al. [9] | Privacy concern, perceived personalization, trust | attribute–perception–intention model | mHealth services acceptance |
| 10 | Hoque [10] | Perceived usefulness, perceived ease of use, subjective norm, personal innovativeness in IT. | TAM | Use mHealth services |
| 11 | Hoque and Sorwar [11] | Performance expectancy, effort expectancy, social influence, facilitating conditions, technology anxiety, resistance to change | UTAUT | Use mHealth |
| 12 | Deng et al. [12] | Perceived value, attitude, perceived behavioral control, subjective norm, perceived physical condition, resistance to change, technology anxiety, self-actualization need | Value attitude behavior model, TPB | Mobile health services |
| 13 | Dam et al. [13] | Positive attitudes, information-seeking gratifications, Social utility gratifications, internal competitiveness orientation, external competitiveness orientation, app competition gratifications, app challenge gratifications | The Integrated Technology Adoption Model | Use health and fitness apps |
| 14 | Meng et al.[14] | Trust in offline health services, trust in mHealth services, declining physiological conditions, support from hospital | Trust transfer model | Use mHealth service |
| 15 | Duarte and Pinho [15] | Performance expectancy, effort expectancy, social influence, facilitating conditions, hedonic motivations, price value, habit | UTAUT | mHealth adoption |
| 16 | Alam et al. [16] | Performance expectancy, effort expectancy, social influence, facilitating conditions, perceived reliability, price value | UTAUT | Adopt mHealth services |
| 17 | Zhang et al. [17] | Perceived severity of the disease, perceived vulnerability to the disease, response cost, response efficacy, attitude, self-efficacy, personal health status, personal health value, subjective norm | TPB, Protection motivation theory. | Use mobile health services |

**Reference**

1. Guo X, Sun Y, Wang N, Peng Z, Yan Z. The dark side of elderly acceptance of preventive mobile health services in China. Electronic Markets. 2013;23(1):49-61.

2. Rai A, Chen L, Pye J, Baird A. Understanding determinants of consumer mobile health usage intentions, assimilation, and channel preferences. Journal of Medical Internet Research. 2013;15(8):e149.

3. Cocosila M. Role of user a priori attitude in the acceptance of mobile health: an empirical investigation. Electronic Markets. 2013;23(1):15-27.

4. Sun Y, Wang N, Guo X, Peng Z. Understanding the acceptance of mobile health services: a comparison and integration of alternative models. Journal of Electronic Commerce Research. 2013;14(2):183-200.

5. Zhang X, Guo X, Lai K-h, Guo F, Li C. Understanding gender differences in m-health adoption: a modified theory of reasoned action model. Telemedicine and e-Health. 2014;20(1):39-46.

6. Shareef MA, Kumar V, Kumar U. Predicting mobile health adoption behaviour: A demand side perspective. Journal of Customer Behaviour. 2014;13(3):187-205.

7. Okazaki S, Blas SS, Castañeda JA. Physicians’ Adoption of Mobile Health Monitoring Systems in Spain: Competing Models and Impact of Prior Experience. Journal of Electronic Commerce Research. 2015;16(3):194-217.

8. Dwivedi YK, Shareef MA, Simintiras AC, Lal B, Weerakkody V. A generalised adoption model for services: A cross-country comparison of mobile health (m-health). Government Information Quarterly. 2016;33(1):174-87.

9. Guo X, Zhang X, Sun Y. The privacy–personalization paradox in mHealth services acceptance of different age groups. Electronic Commerce Research and Applications. 2016;16:55-65.

10. Hoque MR. An empirical study of mHealth adoption in a developing country: the moderating effect of gender concern. BMC Medical Informatics and Decision Making. 2016;16(1):51.

11. Hoque R, Sorwar G. Understanding factors influencing the adoption of mHealth by the elderly: An extension of the UTAUT model. International Journal of Medical Informatics. 2017;101:75-84.

12. Deng Z, Mo X, Liu S. Comparison of the middle-aged and older users’ adoption of mobile health services in China. International journal of Medical Informatics. 2014;83(3):210-24.

13. Dam L, Roy D, Atkin DJ, Rogers D. Applying an integrative technology adoption paradigm to health app adoption and use. Journal of Broadcasting & Electronic Media. 2018;62(4):654-72.

14. Meng F, Guo X, Peng Z, Lai K-H, Zhao X. Investigating the adoption of mobile health services by elderly users: Trust transfer model and survey study. JMIR mHealth and uHealth. 2019;7(1):e12269.

15. Duarte P, Pinho JC. A mixed methods UTAUT2-based approach to assess mobile health adoption. Journal of Business Research. 2019;102:140-50.

16. Alam MZ, Hoque MR, Hu W, Barua Z. Factors influencing the adoption of mHealth services in a developing country: A patient-centric study. International Journal of Information Management. 2020;50:128-43.

17. Zhang X, Liu S, Wang L, Zhang Y, Wang J. Mobile health service adoption in China. Online Information Review. 2019;44(1):1-23.
