# Supplementary material for: Active Usage of Mobile Health Applications: Cross-sectional Study
Source: J Med Internet Res. 2021 Dec 22;23(12):e25330. doi: 10.2196/25330 (PMC8734924; doi:10.2196/25330)
Supplement: Multimedia Appendix 2 [file jmir_v23i12e25330_app2.docx]

# Appendix 2. Literature review of active usage

To understand the literature of active usage of digital applications systematically and comprehensively, we survey previous literature and present the result in table B. We summarize previous literature from four aspects: authors, factors, theories and adoption/usage. According to table 2, many studies focuses on the active usage of different social media and different factors including personal, social, behavioral and cognitive factors are considered. Therefore, previous literature about active usage pays less attention on the active usage of mobile health and it is necessary to study the active usage of mobile health.

| **Table 2. Literature review of active usage** | | | | |
| --- | --- | --- | --- | --- |
| No. | Authors | Factors | Theories | Adoption/usage |
| 1 | Emmanouilides and Hammond [1] | Time since first use, location of use, specific services used | N/A | Active usage of internet |
| 2 | Chen [2] | Connection, frequency usage of Twitter functions. | Uses and Gratifications theory | Active Twitter use |
| 3 | Pagani et al. [3] | Innovativeness, self-identity, social identity | N/A | Active usage of social networking sites |
| 4 | Pagani and Mirabello [4] | Personal engagement, social interactive engagement | Goals and mental models | Active usage of social TV web sites |
| 5 | Davenport et al [5] | narcissism | N/A | Active usage of social media platforms |
| 6 | Kang et al. [6] | Functional benefits, social psychological benefits, hedonic benefits, monetary benefits | Social capital theory, social identity theory, incentive motivation theory | Active participation in restaurant Facebook fan pages |
| 7 | Khansa et al [7] | Incentives, level, tenure, current behavior, prior behavior | Goal setting theory | Active participation in online question-and-answer communities |
| 8 | Gharib et al [8] | Trusting beliefs, affective commitment, generalized reciprocity, information quality, system quality, service quality | Social exchange theory, information systems success model | Active participation in B2B online communities |
| 9 | Pagani and Malacarne [9] | Personal engagement, social interactive engagement, privacy | goals and mental model | Active behavior in mobile location-based social networks |
| 10 | Wu et al. [10] | MIM identification, self-congruence, design aesthetics, interactivity, mobility, feedback | Attachment theory | Active usage of mobile instant messaging applications |
| 11 | Zhang and Jung [11] | Information needs, building self-agency, social interaction, self-expression | Use and gratification theory | Active engagement of health information on WeChat |

**Reference**

1. Emmanouilides C, Hammond K. Internet usage: Predictors of active users and frequency of use. Journal of Interactive Marketing. 2000;14(2):17-32.

2. Chen GM. Tweet this: A uses and gratifications perspective on how active Twitter use gratifies a need to connect with others. Computers in Human Behavior. 2011;27(2):755-62.

3. Pagani M, Hofacker CF, Goldsmith RE. The influence of personality on active and passive use of social networking sites. Psychology & Marketing. 2011;28(5):441-56.

4. Pagani M, Mirabello A. The influence of personal and social-interactive engagement in social TV web sites. International Journal of Electronic Commerce. 2011;16(2):41-68.

5. Davenport SW, Bergman SM, Bergman JZ, Fearrington ME. Twitter versus Facebook: Exploring the role of narcissism in the motives and usage of different social media platforms. Computers in Human Behavior. 2014;32:212-20.

6. Kang J, Tang L, Fiore AM. Enhancing consumer–brand relationships on restaurant Facebook fan pages: Maximizing consumer benefits and increasing active participation. International Journal of Hospitality Management. 2014;36:145-55.

7. Khansa L, Ma X, Liginlal D, Kim SS. Understanding members’ active participation in online question-and-answer communities: A theory and empirical analysis. Journal of Management Information Systems. 2015;32(2):162-203.

8. Gharib RK, Philpott E, Duan Y. Factors affecting active participation in B2B online communities: An empirical investigation. Information & Management. 2017;54(4):516-30.

9. Pagani M, Malacarne G. Experiential engagement and active vs. passive behavior in mobile location-based social networks: the moderating role of privacy. Journal of Interactive Marketing. 2017;37:133-48.

10. Wu T, Lu Y, Gong X, Gupta S. A study of active usage of mobile instant messaging application: An attachment theory perspective. Information Development. 2017;33(2):153-68.

11. Zhang L, Jung EH. WeChatting for health: An examination of the relationship between motivations and active engagement. Health Communication. 2019;34(14):1764-74.
