# Supplementary material for: Active Usage of Mobile Health Applications: Cross-sectional Study
Source: J Med Internet Res. 2021 Dec 22;23(12):e25330. doi: 10.2196/25330 (PMC8734924; doi:10.2196/25330)
Supplement: Multimedia Appendix 3 [file jmir_v23i12e25330_app3.docx]

# Appendix 3. Survey instrument

|  | |
| --- | --- |
| Constructs and sources | Items |
| Active usage (Pagani and Mirabello 2011) | - I spend a lot of time in using mobile health applications. - I frequently interact with doctors and my friends by using mobile health applications. - I post comments/feedbacks of content I view through mobile health applications. |
| Satisfaction (Taylor and Baker 1994) | - I am satisfied with mobile health applications usage. - I am pleased with mobile health applications usage. - Using mobile health applications is usually a satisfying experience |
| Dissatisfaction (Babin and Griffin 1998) | - I felt some dissatisfaction based on my experience with using mobile health applications. - Using mobile health applications is not a wise option. - I am unhappy about using mobile health applications. |
| Sociability (Animesh et al. 2011) | - Mobile health applications enable me to get a good impression of doctors and my friends. - Mobile health applications enable me to develop good social relationships with doctors and my friends. - Mobile health applications enable me to form close friendships with doctors and my friends. |
| Mobility (Hong et al. 2008) | - I can use mobile health applications anytime and anywhere - I find mobile health applications are easy to use and portable - Mobile health applications are available to use whenever I need it. - In general, I have control over using mobile health applications anytime and anywhere. |
| Customization (Srinivasan et al. 2002) | - The mobile health application I used is tailor-made for me. - I believe that the mobile health application I use is customized to my needs. |
| Design aesthetics (Lavie and Tractinsky 2004) | - The interface design of mobile health applications is aesthetic. - The interface design of mobile health applications is clean. - The interface design of mobile health applications is creative. - The interface design of mobile health applications is sophisticated. - The interface design of mobile health applications is pleasant. |
| Perceived security (Cheung and Lee 2006) | - In general, I feel secure in using mobile health applications. - I feel safe in communicating, commenting/posting, or transacting using mobile health applications. |
| Enjoyment (Sun and Zhang 2006) | - I find using mobile applications are to be enjoyable. - The actual process of using mobile applications is pleasant. - I have fun using mobile health applications. |
| Information quality (Ou and Sia 2010) | - The information in mobile health applications appears to be accurate. - The information in mobile health applications appears to be helpful. |
| Informational support (Liang et al. 2011) | - By using mobile health applications, doctors and my friends can give me suggestions when I need help. - When I encountered health problems, doctors and my friends can give me information help me overcome the problems through mobile health applications. |
| Emotional support (Liang et al. 2011) | - When I faced with health problems, doctors and my friends using mobile health applications are on my side. - When I faced with health problems, doctors and my friends using mobile health applications express their interest and concern on my wellbeing. |
| Technical functionality (Ou and Sia 2010) | - Mobile health applications are stable during my usage. - The response time for mobile health applications to process my request is acceptable. - Generally speaking, mobile health applications operate smoothly. |
| Note: Scale ranged from 1 (strongly disagree) to 7 (strongly agree). | |

**Reference**

Animesh, A., Pinsonneault, A., Yang, S.-B., and Oh, W. 2011. "An Odyssey into Virtual Worlds: Exploring the Impacts of Technological and Spatial Environments on Intention to Purchase Virtual Products," *MIS Quarterly* (35:3), pp. 789-810.

Babin, B. J., and Griffin, M. 1998. "The Nature of Satisfaction: An Updated Examination and Analysis," *Journal of Business Research* (41:2), pp. 127-136.

Cheung, C. M., and Lee, M. K. 2006. "Understanding Consumer Trust in Internet Shopping: A Multidisciplinary Approach," *Journal of the American Society for Information Science and Technology* (57:4), pp. 479-492.

Hong, S.-J., Thong, J. Y., Moon, J.-Y., and Tam, K.-Y. 2008. "Understanding the Behavior of Mobile Data Services Consumers," *Information Systems Frontiers* (10:4), pp. 431-445.

Lavie, T., and Tractinsky, N. 2004. "Assessing Dimensions of Perceived Visual Aesthetics of Web Sites," *International Journal of Human-Computer Studies* (60:3), pp. 269-298.

Liang, T.-P., Ho, Y.-T., Li, Y.-W., and Turban, E. 2011. "What Drives Social Commerce: The Role of Social Support and Relationship Quality," *International Journal of Electronic Commerce* (16:2), pp. 69-90.

Ou, C. X., and Sia, C. L. 2010. "Consumer Trust and Distrust: An Issue of Website Design," *International Journal of Human-Computer Studies* (68:12), pp. 913-934.

Pagani, M., and Mirabello, A. 2011. "The Influence of Personal and Social-Interactive Engagement in Social Tv Web Sites," *International Journal of Electronic Commerce* (16:2), pp. 41-68.

Srinivasan, S. S., Anderson, R., and Ponnavolu, K. 2002. "Customer Loyalty in E-Commerce: An Exploration of Its Antecedents and Consequences," *Journal of Retailing* (78:1), pp. 41-50.

Sun, H., and Zhang, P. 2006. "Causal Relationships between Perceived Enjoyment and Perceived Ease of Use: An Alternative Approach," *Journal of the Association for Information Systems* (7:9), pp. 618-645.

Taylor, S. A., and Baker, T. L. 1994. "An Assessment of the Relationship between Service Quality and Customer Satisfaction in the Formation of Consumers' Purchase Intentions," *Journal of retailing* (70:2), pp. 163-178.
